# ASSOCIATIONS BETWEEN NEIGHBORHOOD SOCIAL DESTINATIONS AND GPS-DERIVED COMMUNITY MOBILITY OF OLDER ADULTS

**DOI:** 10.1093/geroni/igad104.1502

**Published:** 2023-12-21

**Authors:** Kyle Moored, Breanna Crane, Pamela Dunlap, Michelle Carlson, Andrea Rosso

**Affiliations:** Johns Hopkins Bloomberg School of Public Health, Baltimore, Maryland, United States; Johns Hopkins Bloomberg School of Public Health, Baltimore, Maryland, United States; University of Pittsburgh, Pittsburgh, Pennsylvania, United States; Johns Hopkins Bloomberg School of Public Health, Baltimore, Maryland, United States; University of Pittsburgh, Pittsburgh, Pennsylvania, United States

## Abstract

Neighborhood social destinations may encourage greater out-of-home travel (i.e., community mobility) and support independent functioning in later life. We examined associations between quantity of destinations that promote social interactions (e.g., arts/recreation, restaurants, stores, civic/religious institutions) and objective, GPS-derived community mobility. Participants were 146 adults ≥65 years old (Mean=77.0±6.5 years, 68% women) at baseline of a randomized trial to improve walking coordination. Participants carried a GPS device that passively collected real-time location data for 5-7 days. Spatial extent of community mobility was quantified using median standard deviational ellipse area (SDEa, km2) and maximum distance from home (mDOH, km). Duration of community mobility was characterized using percentage of time spent out-of-home (pTOH). Community mobility measures were stratified into tertiles due to positive skew. We used National Establishment Time Series (NETS) classifications to derive a total social destination count at the Census tract-level (Mean=48.9±35.7 social destinations per tract). Ordinal logistic regressions were adjusted for demographics (age, gender, race, education), device, season, and number of comorbidities and were clustered on Census tract. Each 1-SD greater destination count was associated with 46% greater odds of being in a higher tertile of pTOH (OR=1.46, 95% CI: 1.08-1.97, p=.014). Number of social destinations was not associated with SDEa or mDOH (all p’s>.05). Nearby social destinations may encourage greater duration, but not necessarily spatial extent, of community travel. Future work utilizing GPS can objectively measure activity in community social spaces to assess its benefits for cognitive and mental health in later life.

